# The secretion of the bacterial phytase PHY‐US417 by *Arabidopsis* roots reveals its potential for increasing phosphate acquisition and biomass production during co‐growth

**DOI:** 10.1111/pbi.12552

**Published:** 2016-03-30

**Authors:** Nibras Belgaroui, Pierre Berthomieu, Hatem Rouached, Moez Hanin

**Affiliations:** ^1^ Laboratoire de Biotechnologie et Amélioration des Plantes Centre de Biotechnologie de Sfax Sfax Tunisie; ^2^ Institut National de la Recherche Agronomique Centre National de la Recherche Scientifique Université Montpellier 2 Montpellier SupAgro. Biochimie et Physiologie Moléculaire des Plantes Montpellier Cedex 2 France; ^3^ Institut Supérieur de Biotechnologie Université de Sfax Sfax Tunisie

**Keywords:** phytic acid, phytases, phosphorus, intercropping

## Abstract

Phytic acid (PA) is a major source of inorganic phosphate (Pi) in the soil; however, the plant lacks the capacity to utilize it for Pi nutrition and growth. Microbial phytases constitute a group of enzymes that are able to remobilize Pi from PA. Thus, the use of these phytases to increase the capacity of higher plants to remobilize Pi from PA is of agronomical interest. In the current study, we generate transgenic *Arabidopsis* lines (ePHY) overexpressing an extracellular form of the phytase PHY‐US417 of *Bacillus subtilis*, which are characterized by high levels of secreted phytase activity. In the presence of PA as sole source of Pi, while the wild‐type plants show hallmark of Pi deficiency phenotypes, including the induction of the expression of Pi starvation‐induced genes (PSI, e.g. *PHT1;4*) and the inhibition of growth capacity, the ePHY overexpressing lines show a higher biomass production and no PSI induction. Interestingly, when co‐cultured with ePHY overexpressors, wild‐type *Arabidopsis* plants (or tobacco) show repression of the PSI genes, improvement of Pi content and increases in biomass production. In line with these results, mutants in the high‐affinity Pi transporters, namely *pht1;1* and *pht1;1‐1;4*, both fail to accumulate Pi and to grow when co‐cultured with ePHY overexpressors. Taken together, these data demonstrate the potential of secreted phytases in improving the Pi content and enhancing growth of not only the transgenic lines but also the neighbouring plants.

## Introduction

Plant growth and crop productivity are largely hindered by the low availability of phosphorus (P) in soil. Plants can only assimilate P as inorganic phosphate (Pi) forms H_2_PO_4_^‐^ and HPO_4_^2‐^, which occur in soil solutions at very low concentrations 0.1–10 μm (Hinsinger, 2001). Therefore, intensive use of P fertilizers was largely followed by farmers to prevent P deficiency and increase agricultural yields (Gilbert, 2009). However, only 10%–20% of fertilizer P is available for utilization by crops in the first year after application (Holford, 1997). The majority of the added P is transformed into inorganic and organic forms which are of limited availability to plants (Sanyal and De Datta, 1991). Between 30 and 80% of the total P in soils exist as organic phosphate (Po), of which up to 60–80% is inositol hexa*kis*phosphate, also known as phytic acid or phytate (PA) that is not directly available to plants (Dalal, 1977; Schachtman *et al*., 1998).

To enhance their ability to obtain adequate P from soil under P limiting conditions, plants have developed a variety of mechanisms, including changes in root morphology and architecture, up‐regulation of high‐affinity phosphate transporters, improvement of internal phosphatase activity and secretion of organic acids and phosphatases (Baker *et al*., 2015; Schachtman *et al*., 1998; Scheible and Rojas‐Triana, 2015; Vance *et al*., 2003). Phosphatases are required for the mineralization of organic P to release Pi into the soil (Raghothama, 1999).

Phytases are special phosphatase enzymes that catalyse the hydrolysis of PA into lower inositol phosphates and Pi. Based on protein structure and catalytic properties, phytases are classified into four distinct families namely histidine acid phosphatases, β‐propeller phytases, purple acid phosphatases and protein tyrosine phosphatase‐like phytases (for review, see Lei *et al*., 2013). Phytases have been detected and characterized in fungi, yeast and bacteria (Lei *et al*., 2007) and have also been found in roots and root exudates of several plants (Hayes *et al*., 2000; Li *et al*., 1997; Richardson *et al*., 2000). However, it has been reported that the enzymatic activity in root exudates is not sufficient for effective utilization of Po (Brinch‐Pedersen *et al*., 2002; Richardson *et al*., 2000).

It is well established that Pi is released from PA following the exogenous application of commercial bacterial phytases in the growth media (Belgaroui *et al*., 2014; Hayes *et al*., 2000; Idriss *et al*., 2002; Richardson *et al*., 2000). Moreover, expression of the intracellular form of β‐propeller phytases from *Bacillus subtilis* in *Arabidopsis* and tobacco was tested and showed an enhancement of the growth performance of these transgenic plants under Pi‐limited conditions (Belgaroui *et al*., 2014; Lung *et al*., 2005). Nevertheless, these approaches had only limited success for implementing strategies to increase crop production under P limitation. In this regard, more attention has been focused on the potential of extracellular form of phytases produced by plant roots for the improvement of organic P use from soil. To date, several genes have been tested using this strategy and led to the improvement of phytate‐P utilization and plant growth in different plant species such as subterranean clover, potato, *Nicotiana tabacum,* cotton, *Brassica napus*, white clover and *Arabidopsis* (George *et al*., 2004, 2005; Liu *et al*., 2011; Ma *et al*., 2009; Wang *et al*., 2013; Xiao *et al*., 2005; Zimmermann *et al*., 2003). However, despite the increasing interest on intercropping, an important agronomical practice defined as the cultivation of two or more crops in proximity, the potential of extracellular phytases to increase the growth of neighbouring plants was not assessed before. In this study, we have generated transgenic *Arabidopsis* that secrete the phytase PHY‐US417 of *Bacillus subtilis* US417. This β‐propeller phytase showed interesting catalytic properties as it exhibits a great pH stability (with an optimum at pH 7.5) and high specificity to PA (Farhat *et al*., 2008). We have set up an experimental system where we demonstrate that the secreted enzyme resulted in promoting growth of not only the transgenic plants but also of neighbouring wild‐type *Arabidopsis* or tobacco plants, grown in medium containing phytate as the sole P source. We provide the evidence that the plant growth promotion of the transgenic and neighbouring plants is due to their capacity to acquire P released from phytate hydrolysis by the secreted PHY US417. Therefore, these data can serve as a basis for implementing an innovative way to maintain plant growth capacity while decreasing the use of P fertilizers by engineering plant‐secreting phytases in intercropping practices.

## Results

### Generation of transgenic *Arabidopsis* plants overexpressing an active form of the extracellular PHY‐US417 phytase

In a recent study (Belgaroui *et al*., 2014), we have established that *Arabidopsis* plants are able to produce an active intracellular form of the phytase PHY‐US417, leading to changes in the response to Pi signalling pathway. As aforementioned, there is a growing interest for the use of secreted phytases not only to better utilize extracellular PA but also for intercropping practices. Therefore, transgenic plants expressing the PHY‐US417 open reading frame fused to the AtExt3 peptide signal sequence (*ePHY‐*US417) were generated (Figure S1A). Molecular and genetic analyses of a number of transformants led to the identification of several T3 independent transgenic lines that are homozygous for HPT (marker gene) and *ePHY‐*US417 genes (Figure S1B‐D). Three transgenic lines named hereafter e4, e5 and e9 were chosen for further analyses.

**Figure 1:**
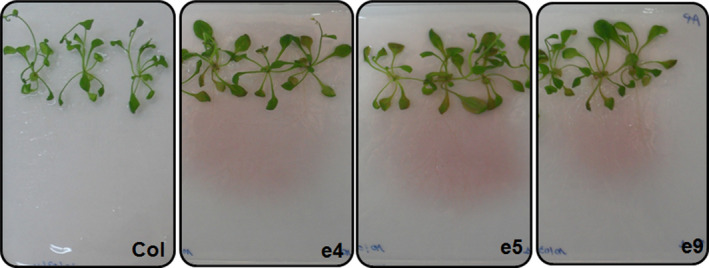
Detection of phytase activity secreted by the roots of ePHY transgenic lines. Three‐week‐old seedlings of three ePHY lines (e4, e5 and e9) together with wild‐type (Col) plants were grown on MS agar plates containing PA as sole P source and stained with FeCl_3_. Pink staining around the roots indicates the absence of PA in the media.

To evaluate the phytase activity of root‐secreted PHY‐US417 on the three selected transgenic lines, two distinct assays were performed. In the first assay, seedlings were germinated on low‐P MS plates containing PA as a sole P source (MS‐P**+**PA), and then, the plates were stained with 0.03% FeCl_3_ as described by Latta and Eskin (1980). As shown in Figure 1, a pink staining could be detected around the roots of the transgenic lines but not of the control plants, revealing the hydrolysis of PA. The second assay consisted of measuring the extracellular phytase activities from exudates of 10‐day‐old seedlings grown on MS‐P+PA medium. Results showed that in contrast to WT plants, high levels of secreted phytase activities (ranging from 27 to 39 mU/μg) were registered in exudates collected from e4, e5 and e9 transgenic lines (Table 1). All these data confirm that the PHY‐US417 secreted by roots of the transgenic lines is active and able to release Pi from PA supplied to the growth medium.

**Table 1:** Extracellular phytase activity and Pi content in transgenic Arabidopsis lines grown in sterile nutrient solution. Phytase activity was determined between day 10 and day 12 of growth. Data are presented as means ± SD of three individual replicates. For each parameter, the values marked by different letters (a and b) are significantly different (*P* < 0.05)

	Secreted phytase activity mU/μg secreted protein	Pi (μm)
Col	0.1 ± 0.01^a^	34.25 ± 1.06^a^
e4	34 ± 6^b^	166 ± 2.82^b^
e5	39.6 ± 4.2^b^	166.5 ± 2^b^
e9	27.4 ± 3.1^b^	120 ± 1.41^b^

### The *ePHY‐US417* overexpressing lines exhibited improved growth and Pi contents under Pi‐limited conditions

**Figure 2:**
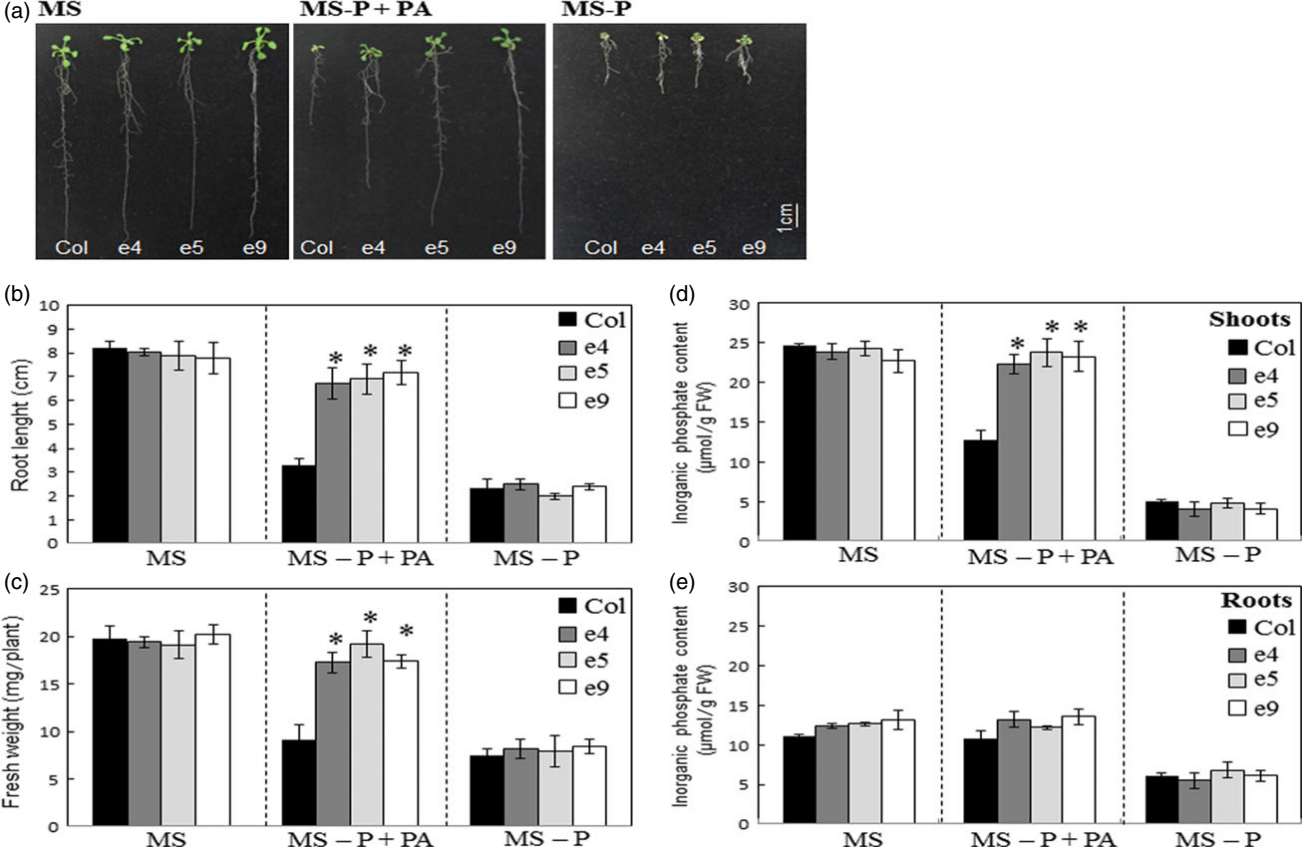
The ePHY transgenic lines maintain their growth on media containing PA as sole P source. (a) Photographs of representative wild‐type (Col) and transgenic (e4, e5 and e9) seedlings were taken after 2 weeks of growth on three types of media: MS (with 1 mm Pi), MS‐P or MS‐P+PA (0.33 mm). (b), (c), (d) and (e) Root lengths, seedling fresh weights and Pi concentrations in roots and shoots. All measurements were performed on WT and transgenic plants grown under growth conditions indicated in (a). Data are means ± SD of three replicates (*n* = 6). For determination of fresh weights and Pi concentrations, pools of 6 plants were used in each replicate. All measurements were performed on 2‐week‐old seedlings. Asterisks indicate a statistically significant difference (*P* < 0.05) with control plants.

The growth rates of the ePHY US417 overexpressing lines were assessed on either MS or MS‐P+PA media for 2 weeks. Under such Pi‐limited conditions, the growth of wild‐type control plants is significantly decreased compared to those grown on MS medium. By contrast, the transgenic lines (e4, e5 and e9) were only slightly affected when PA was supplied instead of Pi (Figure 2a) and showed longer roots (>2‐fold) and higher biomass levels (>2‐fold), compared to control plants (Figure 2b,c). Consistently, when grown on MS‐P+PA medium, all three transgenic lines accumulated more Pi in their shoots than the wild‐type plants. Interestingly, the Pi accumulation reached levels similar to those registered under +Pi conditions (MS medium) (Figure 2d), indicating that they are readily able to obtain Pi from PA. No significant differences in growth were measured between transgenic lines and wild‐type plants, under either ‐Pi (MS‐P) or +Pi (MS) treatments. These results indicate that root secretion of the phytase PHY‐US417 maintains growth and Pi nutrition of *Arabidopsis* transgenic plants on MS medium containing PA as sole P source.

### Secretion of phytase from the roots of ePHY transgenic lines promotes growth of co‐cultivated plants under Pi‐limited conditions

**Figure 3:**
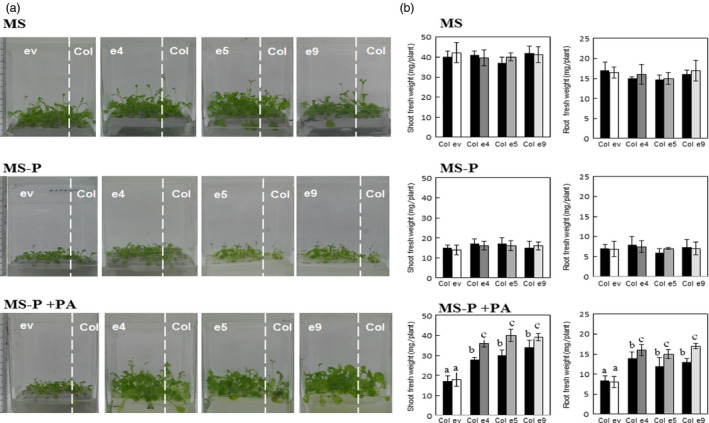
Growth performance of Arabidopsis wild‐type plants co‐cultivated with ePHY overexpressing lines in Pi‐deficient MS medium supplied with phytate. Seven‐day‐old seedlings of wild type (Col), a transgenic line harbouring empty vector (ev) or ePHY overexpressors (e4, e5 and e9) were germinated on MS‐P agar medium, transferred together as indicated, to 50 ml liquid MS (with 1 mm Pi), MS‐P or MS‐P+PA (0.33 mm) and grown for further 2 weeks. Seedlings of WT were co‐cultivated with Ev or ePHY lines using a seedling ratio (Col:ev or Col:ePHY) of 1:4. (a), (b) Phenotypes and root and shoot fresh weights of WT plants and transgenic plants co‐cultivated in MS, MS‐P or MS‐P+PA. Values shown represent mean ± SD of three individual replicates (*n* = 6). Values marked with different letters are significantly different *P* < 0.05, ANOVA.

**Figure 4:**
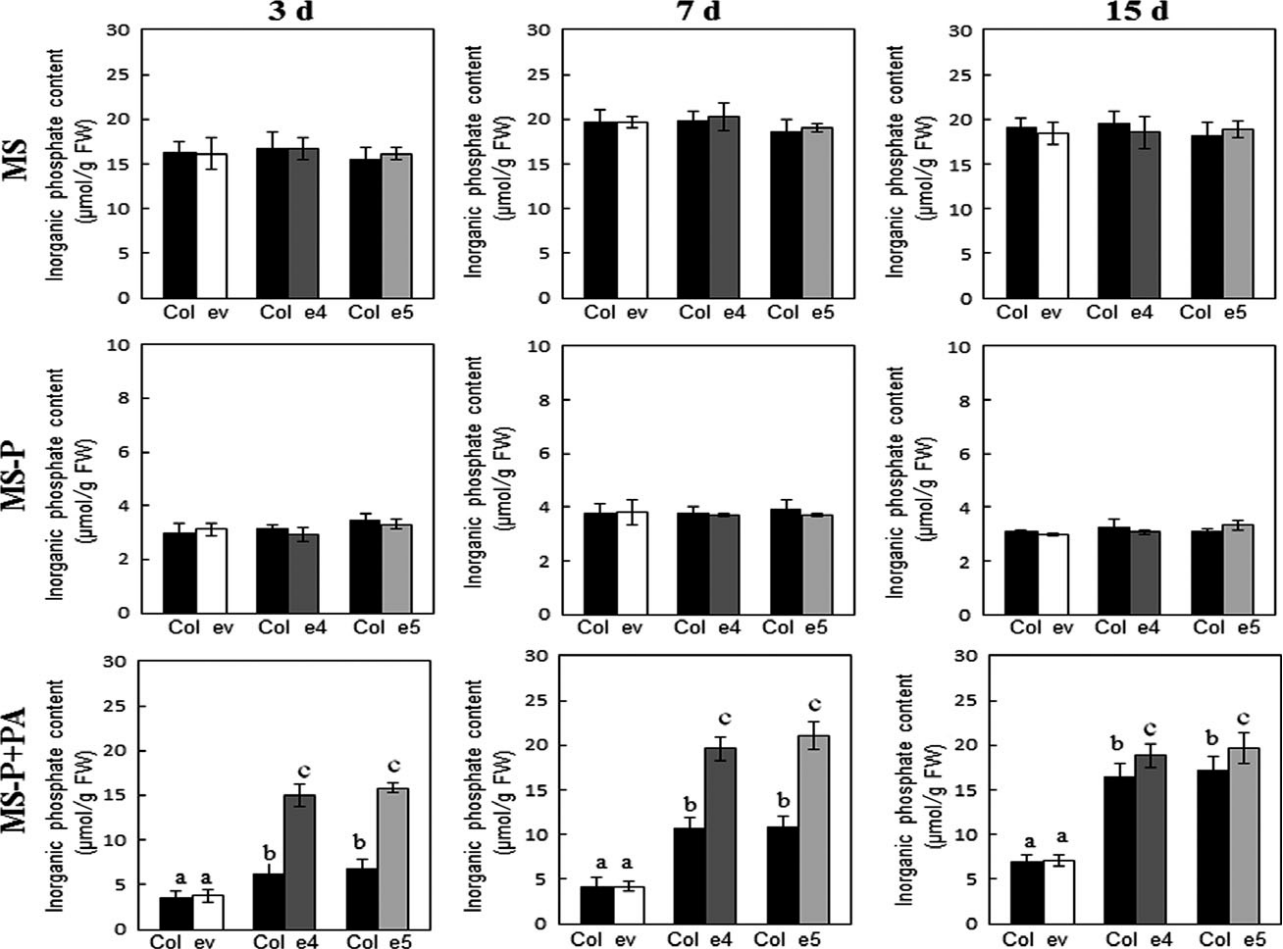
Pi concentrations increase in shoots of WT seedlings when grown together with ePHY lines in medium containing phytate as sole P source. Seven‐day‐old WT seedlings were co‐transferred to liquid MS, MS‐P or MS‐P+PA together with ev line or ePHY overexpressors (e4 and e5) as indicated in Figure 3. Seedlings were harvested 3, 7 and 15 days of transfer to liquid media, and Pi concentrations in shoots were quantified. Data are mean ± SD of three individual replicates (*n* = 6). Values marked with different letters are significantly different *P* < 0.05, ANOVA.

As abovementioned, secretion of the phytase PHY‐US417 from plant roots improved the ability of the transgenic plants to acquire Pi from PA. This finding prompted us to test whether the secreted phytase can also contribute in promoting the growth of other plants co‐cultivated with the transgenic lines under Pi‐limited conditions. For this reason, the ePHY transgenic lines (e4, e5 and e9) and wild‐type *Arabidopsis* seedlings were germinated on MS‐P agar medium and then transferred together with MS‐P+PA liquid medium. Wild‐type plants co‐cultivated with ePHY lines grow far better (with up to ~75% increases in shoot and root fresh weights) than those co‐cultivated with empty vector control plants (ev line) (Figure 3b) and achieve 2 weeks after transfer, high shoots Pi contents, nearly the same registered in the e4 and e5 transgenic lines (Figure 4). Moreover, in these wild‐type plants co‐cultivated with ePHY lines, the Pi uptake increases at rates of 19 ± 2%, 31 ± 1% and 51 ± 2% higher than those co‐cultivated with control plants, after 3, 7 and 15 days of transfer to MS‐P+PA media, respectively (Figure 4). No significant differences in growth and Pi contents (Figures 3 and 4) were observed between wild‐type plants co‐cultivated with transgenic or with ev lines under ‐Pi or +Pi conditions. These results strongly suggest that the growth promotion of co‐cultivated *Arabidopsis* is closely linked to the activity of the phytase secreted by ePHY lines, which is responsible for increasing the Pi concentration in the growth medium.

**Figure 5:**
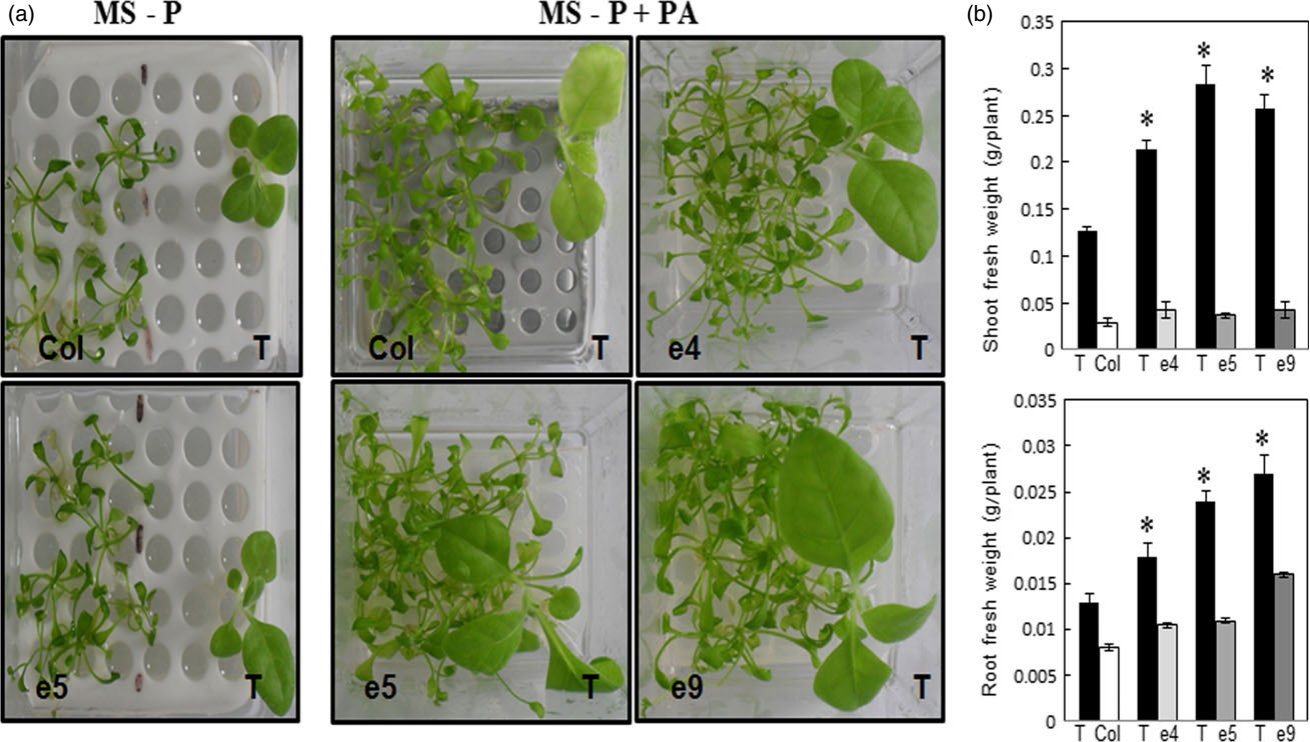
Growth performance of tobacco wild‐type plants co‐cultivated with ePHY overexpressing lines in Pi‐deficient MS medium supplied with phytate. Two‐week‐old tobacco seedlings were transferred together with Col control plants or ePHY transgenic lines to liquid MS‐P or MS‐P+PA (0.33 mm) media. Tobacco seedlings (T) were co‐cultivated in liquid media with WT or ePHY lines using a seedling ratio (T:Col or T:ePHY) of 1:4 for 2 weeks. (a), (b) Phenotypes and root and shoot fresh weights of tobacco seedlings, Arabidopsis WT and transgenic plants co‐cultivated in MS or MS‐P+PA. Data are mean ± SD of three individual replicates (*n* = 6). Asterisks indicate statistically significant difference (*P* < 0.05).

To assess whether the phytase secreted from ePHY overexpressors can promote the growth of co‐cultivated plants other than *Arabidopsis*, similar growth assays were performed where tobacco plants were transferred together with either e4, e5 or e9 to liquid MS‐P media supplemented or not with PA. Under Pi‐limiting conditions, the growth of tobacco seedlings, co‐cultivated with control plants or transgenic lines, was significantly affected. With the addition of PA in the growth medium, tobacco co‐cultivated with wild‐type *Arabidopsis* plants showed a strong inhibition of growth with chlorosis symptoms. This may be related to the unavailability of not only Pi but also other minerals (such as Ca, Zn, Mg and Fe) due to the chelating effect of PA. However, tobacco plants co‐cultivated with ePHY lines showed higher growth rates (Figure 5a) as well as shoot (from 1.6 to 2.2‐fold) and root (from 1.5‐ to 2‐fold) fresh weights (Figure 5b) compared to those co‐cultivated with WT plants. Collectively, these prove that the secreted phytase improves the growth of tobacco neighbouring plants cultivated in the presence of PA as sole source of Pi.

### The ePHY overexpressors promote Pi accumulation in shoots and growth capacity of neighbouring plants

**Figure 6:**
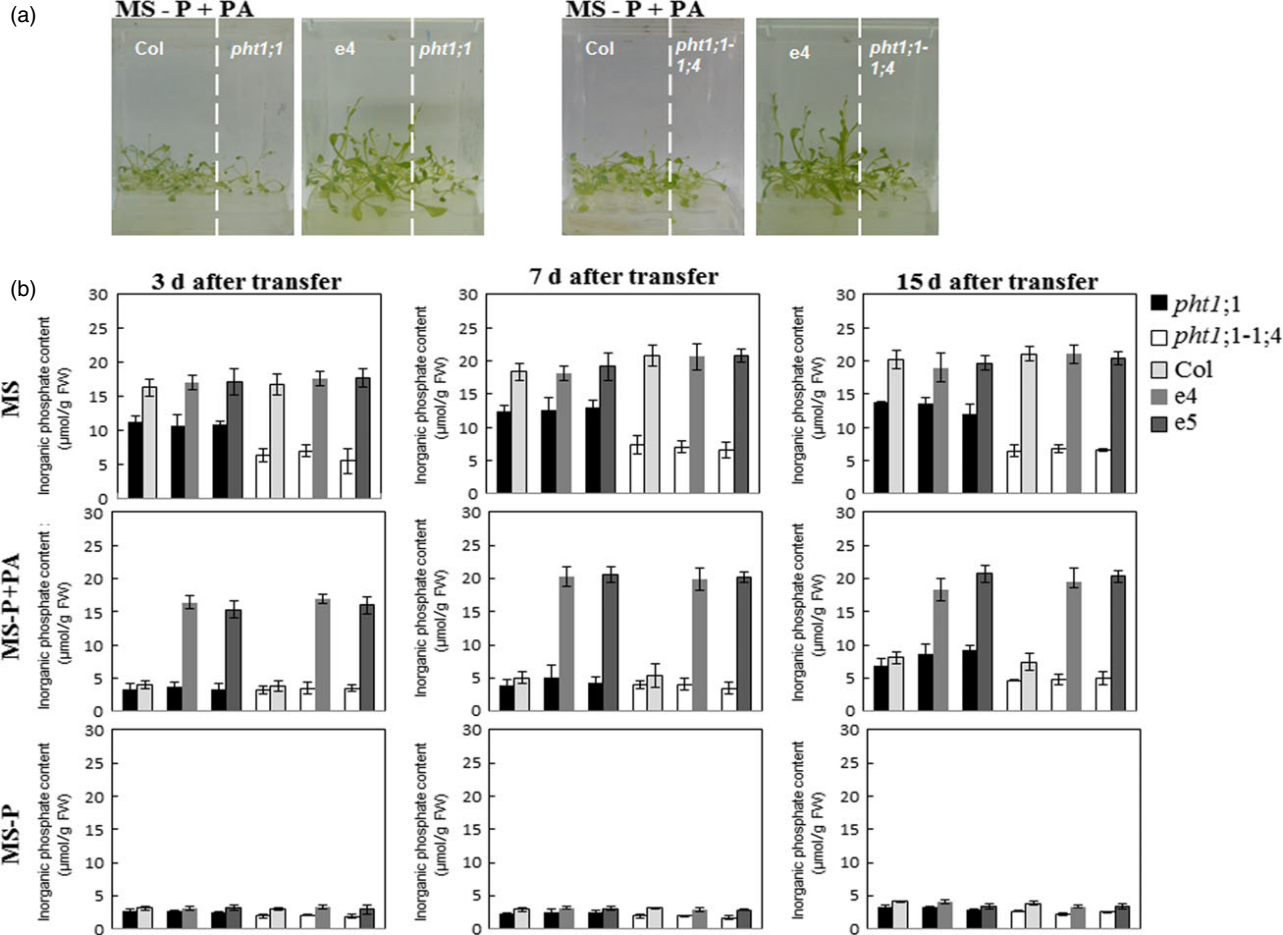
Growth and Pi contents of *pht1;1* and *pht1;1‐1;4* mutants co‐cultivated with wild‐type or ePHY overexpressors. Seven‐day‐old seedlings of wild type (Col), *pht1;1*,* pht1;1‐1;4* or ePHY overexpressors (e4 and e5) were germinated on MS‐P agar medium, transferred together as indicated, to 50 ml liquid MS (with 1 mm Pi), MS‐P or MS‐P+PA (0.33 mm) and grown for further 3, 7 or 15 days. (a) Photographs of representative 2‐week‐old seedlings co‐cultivated in liquid MS‐P+PA under seedling ratio (*pht1;1* or *pht1;1‐1;4*:Col and *pht1;1* or *pht1;1‐1;4*:e4) of 1:4. (b) Pi concentrations in shoots of co‐cultivated plants after 3, 7 and 15 days of transfer to liquid MS, MS‐P and MS‐P+PA media. Data are mean ± SD of three individual replicates (*n* = 6).

The growth promotion of plants co‐cultivated with the ePHY overexpressors in the presence of PA as sole source of P can be most plausibly explained by an increase in Pi availability following PA hydrolysis by PHY‐US417 phytase excreted in the medium and its (Pi) uptake by the plants. To confirm this, two mutants in high‐affinity Pi transporters, *pht1;1* or *pht1;1‐1;4*, which are characterized by a limited Pi uptake capacity, were used in this work (Shin *et al*., 2004). Under standard growth conditions (MS liquid medium), the Pi contents in shoots are 31% and 68% lower in *pht1;1* and *pht1;1‐1;4,* respectively, compared to transgenic lines or WT control (Figure 6b). The growth capacity of *pht1;1* or *pht1;1‐1;4* mutants was severely restricted when grown under Pi‐limited conditions, regardless co‐cultivated with ePHY overexpressors or not (Figure 6a). Such data confirm that *Arabidopsis* plants are able to grow in the presence of PA as sole source of P only when they take benefits from Pi released via the activity of secreted phytase PHY US417, and therefore, we can rule out the effect of any intermediate components produced during PA hydrolysis. This study shows a potential advantage of using these e‐PHY overexpressors to demonstrate the critical role of Pi transport system during plant co‐growth* *under Pi‐limited conditions.

### Expression of phosphate transporters is down‐regulated in plants co‐cultured with ePHY overexpressors in the presence of PA as sole source of Pi

Because *Arabidopsis* wild‐type plants show an improved growth capacity when co‐cultured with ePHY overexpressors in the presence of PA as sole source of Pi, we were interested to assess the expression of Pi uptake transporters (*PHT1;1* and *PHT1;4*), and the Pi exporters (*PHO1* and *PHO1;H1*) both in wild‐type plants and ePHY overexpressors grown together under this cultural condition, using MS and MS‐P conditions as controls.

**Figure 7:**
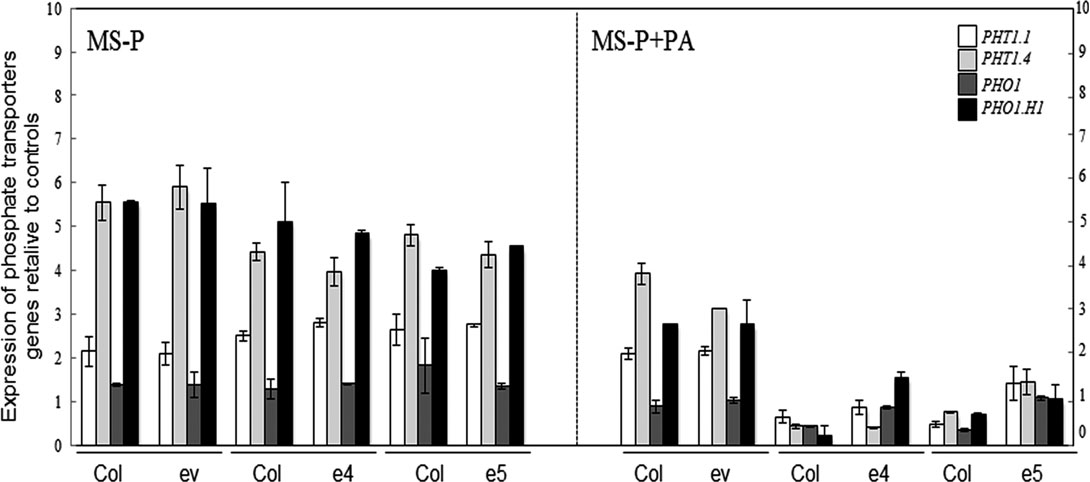
Analysis of the expression of Pi transporters *PHT1;1*, *PHT1;4*, *PHO1* and *PHO1;H1* after co‐growth of *Arabidopsis* plants in liquid MS containing low Pi in the absence (MS‐P), or in the presence of PA (MS‐P+PA) for 2 weeks. WT (Col) was co‐grown hydroponically with either transgenic lines expressing empty vector (ev) or the ePHY overexpressors (e4 and e5). Abundance of *PHT1;1, PHT1;4*, *PHO1* and *PHO1;H1* transcripts was normalized against their respective expression in control condition (MS). Individual measurements were obtained from the analysis of roots collected from a pool of five plants. Data are mean ± SD of three biological replicates.

As expected, in all tested lines (wild‐type plants, ev control line and *ePHY* overexpressors) the expression of these Pi transporters was up‐regulated in response to Pi depletion (MS‐P; Figure 7), compared to MS medium. When grown under MS‐P+PA condition together with ev control line, wild‐type plants showed only a moderate decrease in the expression of all Pi transporters, suggesting that the presence of PA did not alter their Pi deficiency signalling pathway. In contrast, a down‐regulation of these genes was observed in the ePHY overexpressors grown under the same condition, indicating the recovery of the Pi status following the hydrolysis of PA. Most interestingly, when co‐cultured with ePHY overexpressors in MS‐P+PA medium, the wild‐type plants showed also a down‐regulation of the Pi transporters to levels similar to those observed under standard growth conditions. Such a down‐regulation seems to be again due to Pi that becomes more available in the growth medium after PA hydrolysis by the phytase secreted from the roots of ePHY overexpressing lines.

**Figure 8:**
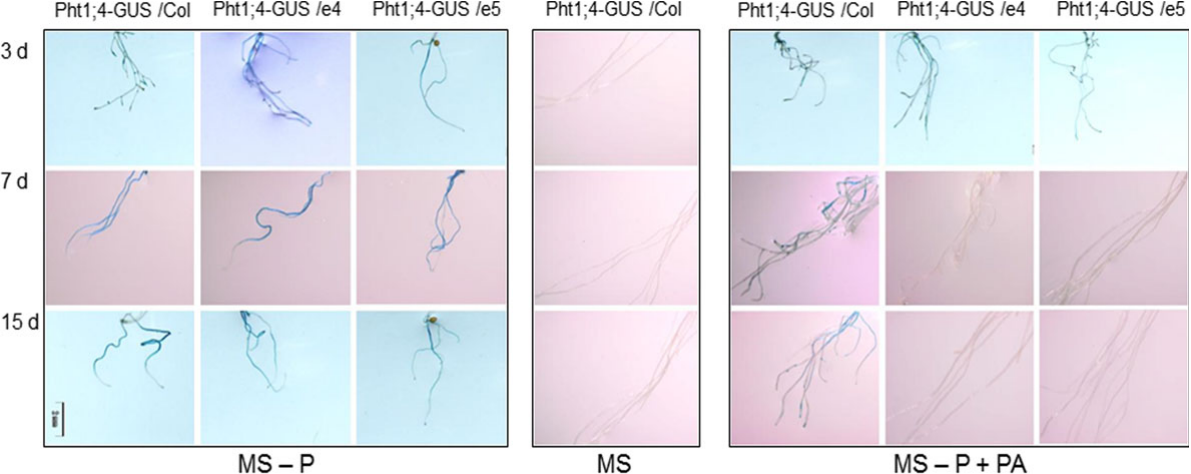
Detection of GUS activity in the PHT1;4::GUS line co‐cultivated with ePHY transgenic line or WT plants. Seven‐day‐old seedlings of the PHT1;4::GUS line were transferred together wild type (Col), or ePHY overexpressors (e4 and e5) to liquid MS (with 1 mm Pi), MS‐P or MS‐P+PA (0.33 mm). GUS staining was performed on the roots of the PHT1;4::GUS line after 3, 7 and 15 days of transfer.

The GUS reporter gene driven by the native promoter of *PHT1;4* (*PHT1;4::GUS*) (Misson *et al*., 2004) was then chosen to infer the expression of the *PHT1;4* gene in roots of this transgenic line co‐cultivated with *ePHY* overexpressors. Seven‐day‐old seedlings of *PHT1;4::GUS* line were transferred with ePHY transgenic lines or with wild‐type *Arabidopsis* plants into hydroponic culture for two additional weeks. The expression of *PHT1;4::GUS* was detected after 3, 7 and 15 days of transfer together with WT plants to MS‐P+PA or to MS‐P. When co‐transferred with ePHY overexpressors, similar GUS staining was detected but only on MS‐P medium. On MS‐P+PA, the roots of *PHT1;4::GUS* line showed only a weak GUS activity at 3 days after transfer, but not at 7 or 15 days after transfer (Figure 8). These results indicate that the Pi deficiency signal was repressed following a substantial increase in Pi concentrations in the growth medium, which is taken up by the plants. This finding provides additional evidence showing that Pi release from PA could be sensed and uptaken efficiently by neighbouring plants.

## Discussion

The world's main source of P is phosphate (Pi) rock. Pi in fertilizers comes from P‐rich mines, and there will be no other economically viable solution once these P‐rich deposits are fully exploited. Knowing that the majority of Pi is associated to PA in soils and that plants cannot use this organic form because of their weak extracellular phytase activity (Hayes *et al*., 2000; Richardson *et al*., 2000), alternative approaches to use the PA are urgently needed. As aforementioned, different strategies have been proposed ranging from the supply of growth medium with microbial phytases (purified mainly from *Aspergillus niger* or *Bacillus subtilis*), to the generation of plants expressing and secreting phytases in root‐medium interface. These strategies were successfully established, but are limited to the benefit of individual plants (Belgaroui *et al*., 2014; Richardson *et al*., 2001), and until now, an eventual benefit for plants grown in proximity was not investigated. So far, the use of secreted microbial phytases to explore new growing strategies such as intercropping was never reported. In this context, the present work shows (i) that *Arabidospsis* plants are able to produce and secrete a functional *Bacillus subtilis* phytase PHY‐US417, which in turn improves plant growth in the presence of PA as sole source of P; (ii) the involvement of high‐affinity transport system, namely PHT1;1 and PHT1;4, in the uptake and accumulation of Pi released by PHY‐US417‐mediated PA hydrolysis; (iii) and most importantly, plant‐secreting microbial phytases have the potential to enhance growth of neighbouring plants under Pi‐limited conditions.

*Arabidopsis* is not able to use PA as source of Pi, and plants undergo Pi deficiency stress as revealed by the induction of the expression of Pi starvation response gene. In the presence of PA, the transgenic plants (e4, e5 and e9 lines) showed a recovery from Pi deficiency stress and exhibit a better growth capacity. These results are consistent with earlier works showing transgenic *Arabidopsis* and tobacco plants secreting microbial phytases exhibit a better growth (Lung *et al*., 2005; Richardson *et al*., 2001).

It is worth noting that to enable the ePHY transgenic lines secreting the ePHY‐US417, they were transformed with a construct containing the coding sequence of this bacterial phytase that is driven by the constitutive CaMV 35S promoter and fused to signal sequence of the *Arabidopsis* extensin gene *AtExt*3. Similar approaches were also previously performed using microbial or plant phytases. Transgenic *Arabidopsis* or tobacco plants constitutively expressing extracellular forms of the fungal phytase *phyA* were engineered using signal peptides from carrot or *Arabidopsis* extensins (George *et al*., 2004, 2005; Lung *et al*., 2005). In other reports, root‐specific promoters were employed to express the fungal phytase in transgenic crop plants, such as cotton and potato (Liu *et al*., 2011; Zimmermann *et al*., 2003). There were also few reports dealing with transgenic plants expressing the phytase (MtPHY1) and a purple acid phosphatase (MtPAP1) genes of *Medicago truncatula*, harbouring their native signal peptides that direct the extracellular secretion of the expressed enzymes (Ma *et al*., 2009; Xiao *et al*., 2005). In all these cases, an improvement in plant Pi acquisition and growth under Pi‐limited conditions was observed. However, again attention was given only to the effect of secreted phytases on P nutrition and growth of the transgenic plants expressing the phytase and never of neighbouring plants. From our findings, we expect that similar positive effects on plant co‐growth can be also observed with other transgenic plants expressing extracellular phytases.

On the other hand, the hydrolysis of PA which releases not only Pi but also other inositol phosphate intermediates posed the question about the role of the hydrolysis products on the growth of the plants. The fact that the high‐affinity Pi transporter mutants *pht1;1* or *pht1;1‐1;4* fail to take benefit from the co‐growth with the ePHY transgenic lines (Figure 6) constitutes a genetic evidence that Pi absorbed is the major factor contributing into improving growth of co‐cultured plants when supplied with PA as sole P source.

Remarkably, Pi contents in *Arabidopsis* wild‐type plants co‐cultivated with the ePHY overexpressors under Pi‐limited conditions remained almost as recorded when grown on complete medium (MS), which involves the high‐affinity Pi transporters to absorb the Pi released from PA by the phytase secreted from ePHY lines. Consistent with this data, Figure 7 shows that the increase of Pi in the growth medium via the hydrolysis of PA by the root‐secreted phytase is sensed by neighbouring plants which in turn down regulates the expression of Pi starvation‐induced genes such as the *PHT*1.4 transporter.

The present study shows for the first time that the phytase PHY‐US417 of *B. subtilis* secreted by the roots of transgenic *Arabidopsis* plants promotes the growth of neighbouring plants, *Arabidopsis* or tobacco, on medium supplied with PA as sole P source. Under such conditions, *Arabidopsis* and tobacco plants showed growth rates similar to those registered under standard conditions. Our findings constitute a promising start for developing novel intercropping practices, and further suited assays would be a plant growth assessment in amended soils with higher PA availability as previously reported (George *et al*., 2005).

Few studies explored the improvement of Pi acquisition and plant growth in intercropping systems. It was shown that chickpea mobilized soil organic P and left more Pi available to the intercropped wheat (Li *et al*., 2003). Moreover, intercropping with faba bean has been reported to improve maize grain yield significantly and above‐ground biomass, compared with maize grown with wheat, at lower rates of P fertilizer application, but not significantly at high rate of P application (Li *et al*., 2007). In general, cereal and legumes represent the most popular combination in intercropping systems. It has been proposed that in most cereal/legume intercropping the cereal shall benefit from the legume species, because legumes are known to excrete larger amounts of carboxylates (Neumann and Römheld, 1999; Pearse *et al*., 2006; Vance *et al*., 2003), phosphatases (Nuruzzaman *et al*., 2006) and mainly protons (Hinsinger *et al*., 2003; Tang *et al*., 1997) in their rhizosphere. In this context, using permeable and impermeable root barriers, Li *et al*. (2007) found that maize overyielding resulted from its uptake of Pi mobilized by the acidification of the rhizosphere via faba bean root release of organic acids and protons.

The present work provides first evidence that engineering plants able to secrete a functional phytase that can release Pi from PA, results in promoting P nutrition and growth of not only the transgenic line but also neighbouring plants. These results pave the way towards implementing innovative intercropping practices as an alternative for sustainable agriculture.

## Experimental procedures

### Plant material, growth conditions and P‐stress treatments

Seeds of tobacco (*Nicotiana tabacum* var Xanthi), *Arabidopsis thaliana* ecotype Columbia (Col‐0), transgenic lines expressing *ePHY‐*US417 (see below), the *pht1;1* and *pht1;1‐1;4* mutants and transgenic lines expressing the GUS reporter gene under the PHT1;4 promoter (Misson *et al*., 2004; Shin *et al*., 2004) were used in these experiments. In all experiments, plant were grown in growth chambers at 22–24°C with a light intensity of 250 μmol/m^2^/s under long‐day conditions 16 h/8 h light/dark cycle. For *in vitro* growth assays, seeds of *Arabidopsis* were germinated and grown in a vertical position for 2 weeks on either MS (Murashige and Skoog, 1962) containing 1 mm Pi KH_2_PO_4_, MS‐P(5 μm KH_2_PO_4_) or MS‐P+PA (5 μm KH_2_PO_4_ +0.33 mm PA). For liquid culture, seedlings were first grown on MS‐P medium for 1 week and then transferred carefully to Magenta boxes containing 50 mL MS, MS‐P or MS‐P+PA media, on an orbital shaker set at 60 rpm. Wild‐type (WT) or mutant plants co‐cultivated with transgenic lines (with a seedling ratio of 1:4) were collected after 3, 7 and 15 days of transfer for phenotypic, physiological and molecular analyses.

### Molecular cloning and generation of transgenic plants

Signal sequence of extensin gene from *Arabidopsis thaliana* (AtExt3) was chosen for directing the secretion of the phytase PHY‐US417 of *Bacillus subtilis* from plant roots. The extensin leader (32 amino acids) of the *AtExt3* gene was amplified from genomic DNA of *A. thaliana* by PCR using the primers Ext3‐Fw5′‐GCTCTAGAATGGCCTCTTTAGTGGCAA‐3′ and Ext3‐Rv5′‐TAGGATCCAGAAGAATAGAAATAGTTAGCGGTTG‐3′. The amplicon was subcloned into pGEM‐T easy cloning vector (Promega) and sequenced. The open reading frame of PHY‐US417, described previously (Belgaroui *et al*., 2014), was amplified by PCR with high‐fidelity Taq polymerase (Stratagene, La Jolla, CA), using the primers PHYC‐BH 5′‐CGGATCCTTATGTCCGATCCTTATCA‐3′(*BamHI* site underlined) and PHYC‐Sph 5′‐GCGCATGCTTATTTTCCGCTTCTGTCGG‐3′(*SphI* site underlined). The PCR product was introduced as a *BamHI*/*SphI* fragment, downstream the *Ext3* leader sequence. Sequence analysis of the resulting construct confirms the in‐frame fusion Ext3‐PHY US417. Then, the fusion was introduced as *SpeI*/*PmlI* fragment into the pCAMBIA1302 binary vector between the cauliflower mosaic virus (CaMV) 35S promoter and the nopaline synthase (NOS) terminator. This binary vector contains also the hygromycin resistance gene (HPT) as a selection marker. The resulting construct pC1302‐Ext3‐PHY US417 (Figure S1A) was checked by sequencing and then used to transform *Arabidopsis thaliana* (ecotype Columbia, Col‐0) plants by floral dipping using *Agrobacterium* strain GV3101 (Clough and Bent, 1998).

Transgenic plants were selected on MS agar medium supplemented with 15 μg/mL hygromycin. From 20 T1 independent transformants, eight T2 lines showing a 3:1 segregation for hygromycin resistance were grown up to T3 generation. Homozygous lines were selected and used in this work.

### Molecular characterization of transgenic lines

Genomic DNA was prepared from leaves of independent transformants and used for PCR screening based on HPT marker as described previously (Belgaroui *et al*., 2014). The presence of *Ext3‐ PHY US417* transgene in the genome of ePHY lines was verified by PCR using Ext3‐Fw (forward) and PHYC‐Sph (reverse) primers.

Total RNA was first extracted from 2‐week‐old seedlings of wild‐type *Arabidopsis* and transgenic lines using Trizol reagent (Invitrogen) and used for RT‐PCR analysis. DNase‐treated RNA samples were reverse‐transcribed using M‐MLV reverse transcriptase (Promega). First‐strand cDNAs were employed as a template for PCR amplifications with a Ext3‐Fw/PHYC‐Sph or HPT‐specific primers (Belgaroui *et al*., 2014). *Actin* gene used as control was amplified with the primers: ActF: 5′GGCGATGAAGCTCAATCCAAACG‐3′ and ActR: 5′GGTCACGACCAGCAAGATCAAGACG‐3′.

### Detection and measurement of secreted phytase activities

Wild‐type and transgenic lines were germinated and grown on agar MS media. Then, 10‐day‐old seedlings were transferred to 125‐ml conical flasks containing the nutrient solution (liquid MS‐P+PA) with agitation (∼60 rpm) for a further 2 days. Extracellular phytase was determined as the amount of activity secreted by seedlings into fresh nutrient solution. Briefly, root‐bath solutions were filtered (0.45 μm) to remove the sloughed‐off cells. An aliquot of root exudates was then incubated with three volumes of MES/Ca buffer (pH 7.5) containing 2 mm myo‐inositol hexa*kis*phosphate (InsP6; Sigma‐Aldrich Corp., St. Louis, MC) as a substrate and incubated at 55°C for 30 min. The reaction was terminated with the addition of an equal volume of 10% trichloroacetic acid (TCA) at either time zero or at the end of incubation. Released Pi was quantified spectrophotometrically at 820 nm using the molybdate blue method (Murphy and Riley, 1962). Enzyme activities were calculated as the difference in phosphate concentrations in supernatants between time zero and the end of incubation. One unit (U) of phytase is the activity that releases 1 mmol of phosphate per minute under these assay conditions. Protein concentrations were determined according to Bradford (1976) using bovine serum albumin as a standard. Pi concentrations were calculated from a standard curve determined using known concentrations of potassium phosphate.

Phytase activity on agar medium containing phytate as sole P source was also visualized by staining of the plates using the wade reagent (0.03% FeCl3‐6H2O and 0,3% sulfosalicylic acid) (Dragičević *et al*., 2011; Latta and Eskin, 1980), following the procedure described by Richardson *et al*. (2001).

### Histochemical GUS staining

Seedlings harbouring the PHT1;4::*GUS* transgene were transferred together with ePHY lines to either MS, MS‐P or MS‐P+PA. Following 3, 7 or 15 days of transfer, a GUS staining was performed on the roots of the PHT1;4::*GUS* line as described by Jefferson *et al*. (1987). Briefly, samples were submerged in a GUS staining solution (50 mm sodium phosphate buffer pH 7.0; 0.5 mm K_3_(Fe[CN]_6_), 0.5 mm K_4_(Fe[CN]_6_), 0.1% Triton X‐100, 1 mg/mL 5‐bromo‐4‐chloro‐3‐indolyl β‐D‐glucuronide cyclohexylammonium salt (X‐Gluc)) and incubated at 37°C overnight. Endogenous pigments including chlorophyll were removed by soaking the plants tissues for several hours in 70% ethanol. Samples were photographed using a Zeiss binocular microscope (Olympus BH‐2, Volketswil, Switzerland).

### Pi measurements

Pi measurements were performed as described by Khan *et al*. (2014). Ion extractions from weighed fresh shoots and roots were performed in water by incubation for 30 min at 70°C. The quantification of Pi was completed by the molybdate assay according to Ames (1966).

### Real‐time quantitative PCR

Total RNA free of residual genomic DNA was prepared from root tissues using the RNeasy extraction kit (Qiagen) and RQ1 RNAse‐free DNAse (Promega). cDNA was synthesized from 2 μg total RNA using an oligo (dT) primer and M‐MLV reverse transcriptase (Promega). Real‐time quantitative reverse‐transcription PCR (qPCR) was performed with a LightCycler 480 Real‐Time PCR System using SYBR green dye technology (Roche) and specific primers as described by Rouached *et al*. (2011). Data were analysed using the Roche LC480 software. Quantification of the relative transcript levels was performed using the comparative CT method (Livak and Schmittgen, 2001; Rouached *et al*., 2008). The relative expression of each gene was normalized to the level of ubiquitin10 transcript (UBQ10: At4 g05320) and expressed as relative values against wild‐type plants grown in complete MS medium.

### Statistical analysis

Analysis of variance (ANOVA) and the Tukey's test were employed to perform statistical analysis presented in this work.

## Conflict of interest

Authors have no conflict of interest to declare.

## Supporting information


**Figure S1.** Generation of transgenic *Arabidopsis* plants overexpressing espy‐US417.
