# Supplementary figures and images for: The secretion of the bacterial phytase PHY‐US417 by Arabidopsis roots reveals its potential for increasing phosphate acquisition and biomass production during co‐growth
Source: Plant Biotechnol J. 2016 Mar 30;14(9):1914–24. doi: 10.1111/pbi.12552 (PMC5071791; doi:10.1111/pbi.12552)

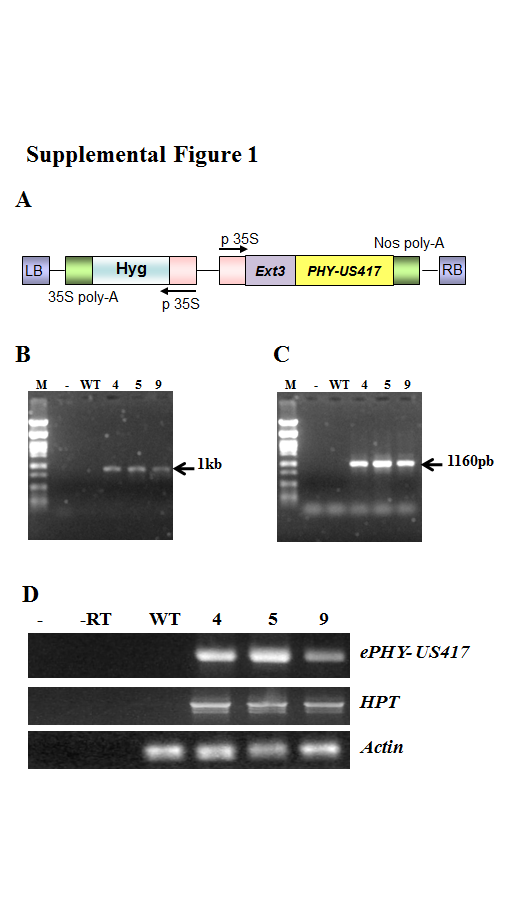

Supplement: Supplementary file 1 — Figure S1. Generation of transgenic Arabidopsis plants overexpressing espy‐US417. [file PBI-14-1914-s001.tiff]
